# Identification of isolates of the plant pathogen *Leptosphaeria maculans* with resistance to the triazole fungicide fluquinconazole using a novel *In Planta* assay

**DOI:** 10.1371/journal.pone.0188106

**Published:** 2017-11-15

**Authors:** Angela P. Van de Wouw, Vicki L. Elliott, Steven Chang, Francisco J. López-Ruiz, Steven J. Marcroft, Alexander Idnurm

**Affiliations:** 1 School of BioSciences, University of Melbourne, Parkville, Victoria, Australia; 2 Marcroft Grains Pathology, Grains Innovation Park, Horsham, Victoria, Australia; 3 Centre for Crop and Disease Management, Department of Environment and Agriculture, Curtin University, Bentley, Western Australia, Australia; New South Wales Department of Primary Industries, AUSTRALIA

## Abstract

*Leptosphaeria maculans* is the major pathogen of canola (oilseed rape, *Brassica napus*) worldwide. In Australia, the use of azole fungicides has contributed to the 50-fold increase in canola production in the last 25 years. However, extensive application of fungicides sets the stage for the selection of fungal populations with resistance. A high-throughput *in planta* assay was developed to allow screening of thousands of isolates from multiple populations. Using this screen, isolates were identified with decreased sensitivity to the fungicide fluquinconazole when applied at field rates as a protective seed dressing: these isolates cause significantly larger lesions on cotyledons and true leaves and increased disease severity at plant maturity. This increased *in planta* resistance was specific to fluquinconazole, with no cross resistance to flutriafol or tebuconazole/prothioconazole. In a limited set of 22 progeny from a cross between resistant and susceptible parents, resistance segregated in a 1:1 ratio, suggesting a single gene is responsible. A survey of 200 populations from across canola growing regions of Australia revealed fungicide resistance was present in 15% of the populations. Although i*n vitro* analysis of the fungicide resistant isolates showed a significant shift in the average EC_50_ compared to the sensitive isolates, this was not as evident as the *in planta* assays. The development of this novel, high-throughput *in planta* assay has led to the identification of the first fungicide resistant *L*. *maculans* isolates, which may pose a threat to the productivity of the Australian canola industry.

## Introduction

The use of fungicides has become an integral part of minimising the impact of plant pathogens in cropping systems. There are currently over 200 fungicides registered for use in food production and they represent 57 different modes of action; however, the market is dominated by chemicals with a small number of modes of action [1]. Three of the most popular classes of chemicals include the demethylation inhibitors (DMI), strobilurins, and succinate dehydrogenase inhibitors (SDHI) [1]. These three classes have single site modes of action that target different pathways in fungal pathogens. The DMI fungicides, also known as azole fungicides, of which the triazoles are a subclass, target the cytochrome P450 enzyme 14α-demethylase encoded by the *Cyp51*/*ERG11* gene [2]. The strobilurin fungicides, also known as Q_o_ inhibitors, act by inhibiting mitochondrial respiration through binding to the Q_o_ site of cytochrome *b* which forms part of the cytochrome *bc*_*1*_ complex [3]. Lastly, the SDHI fungicides target the mitochondrial succinate dehydrogenase (SDH) enzyme, also interfering with mitochondrial respiration [4].

The continual use of fungicides to control fungal pathogens can lead to the selection of resistance, as has already been seen for all the commonly used modes of action [5]. The identification and development of new fungicides is estimated to take around 10 years and approximately $260M USD [1]; therefore, it is essential to minimise and manage the evolution of fungicide resistance to existing chemistries.

There are three main mechanisms of resistance to the triazole fungicides: point mutations within the *Cyp51* (also known as *ERG11*) gene, overexpression of *Cyp51* and overexpression of genes encoding efflux pumps (reviewed by [6]). Point mutations within the *Cyp51* gene are the most widely reported mechanism for resistance towards triazoles, with reports from diverse plant pathogenic fungi including *Zymoseptoria tritici* [7], *Blumeria graminis* f. sp. *tritici* [8], *Mycosphaerella fijiensis* [9] and *Venturia nashicola* [10]. Target site mutations are also the major mechanism for resistance to strobilurins and SDHIs. A range of mutations has been detected within the SDH-encoding genes of fungi which confer resistance to SDHIs [4]. For resistance to strobilurins, there are two amino acid substitutions within the cytochrome b protein that confer resistance in the majority of pathogens; these are the glycine to alanine substitution at position 143 and the phenylalanine to leucine substitution at position 129 [3].

Fungicides, as for many crops, are an important tool in the control of diseases of canola (*Brassica napus*). Blackleg disease is the primary disease of canola worldwide and is caused by the ascomycete *Leptosphaeria maculans*. This disease causes annual losses in Australia of 10–15% with localised epidemics resulting in up to 90% yield loss [11, 12]. Blackleg disease is managed by strategies that include breeding for resistance, farming practises such as avoidance of stubble that is the source of fungal inoculum, rotation with cereals, rotation of canola cultivars with different resistance genes, and the use of fungicides [13–15]. The use of fungicides to control blackleg disease in Australia has dramatically increased since 2005 and has become essential to maintaining current production levels [16]. Three fungicides currently approved and used throughout Australia are fluquinconazole as a seed dressing (registered name Jockey®), flutriafol as a dressing applied with fertiliser (registered name Impact® in Furrow), and a mixture combining prothioconazole and tebuconazole as a foliar application (registered as Prosaro®) [16]. All three of these fungicides belong to the same mode of action class, the azoles. In some situations, all three fungicides are used on a single crop in a single season. In addition, the *L*. *maculans* population may be exposed to other triazole fungicides when applied in the field to other crops being grown in rotation because the current practise of minimal tillage results in the retention of canola stubble, which is infected with *L*. *maculans*, into subsequent years when other crops are in cultivation. The high intensity use of fungicides that all belong to the same class has the potential to select for fungicide resistance in *L*. *maculans* populations. This type of risk has already been realized in canola crops in the United Kingdom, with the extensive use of azole classes of fungicides to control light leaf spot disease leading to the emergence of resistant isolates with point mutations within the *Cyp51* gene of the causative agent, *Pyrenopeziza brassicae* [17].

In this study, we developed a high-throughput assay using fungicide-treated plants to detect the presence of isolates able to overcome the application of triazoles to cause disease. Using this assay, we identify isolates with increased *in planta* resistance to the triazole, fluquinconazole. This segregation of this property in progeny from a genetic crosses is consistent with it being controlled by a single locus. An analysis of 200 populations of *L*. *maculans* from across Australia identified 15% as resistant.

## Materials and methods

### Stubble collection

In the first survey for fungicide resistance, eight stubble samples were collected at the end of the 2013 growing season (S1 Table) and placed on the bare earth in a paddock to allow *L*. *maculans* growing saprotrophically in this material to undergo sexual reproduction and for the pseudothecia (sexual fruiting bodies) to mature over the summer. These eight stubble samples were collected from cultivars from trial sites that had received various applications of fungicide. In June/July of 2014, these stem samples were used to inoculate seedlings treated with fungicide, as described below, to determine if any of these populations could overcome fungicide treatment. Subsequently in a more comprehensive survey in 2015, 200 stubble samples were collected by researchers, agronomists and farmers from across all major Australian canola-growing regions (S1 Table) and used to inoculate seedlings as described below.

### *In planta* screen for fungicide resistance using ascospore showers

**Fig 1:**
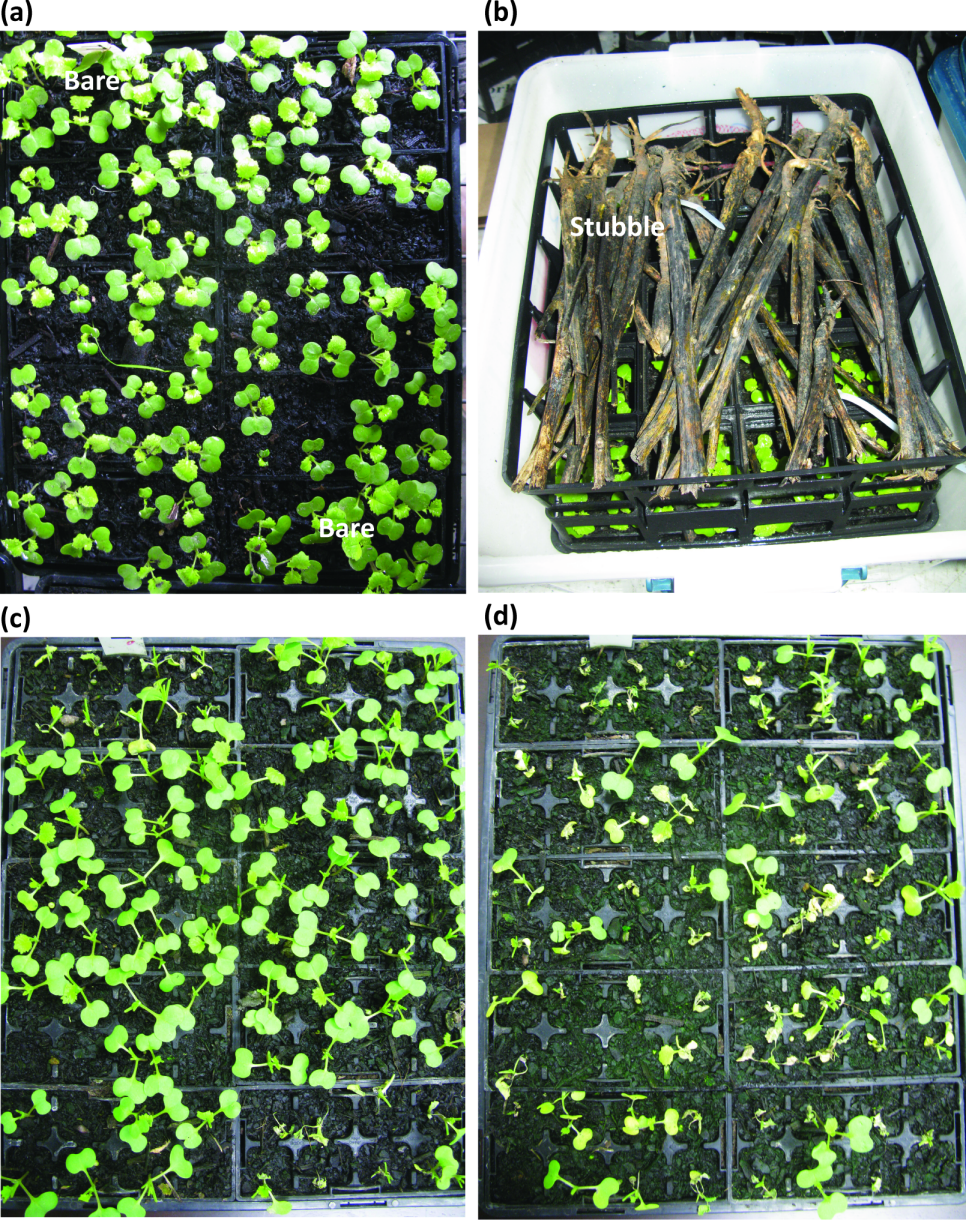
Method for *in planta* screening for resistance to the fungicide fluquinconazole in *Leptosphaeria maculans* populations. (a) Trays containing ten punnets are sown, with the upper left and lower right punnet with bare seed while the remaining eight punnets with seed treated with fluquinconazole. (b) Seedlings are inoculated ten days after sowing using the ascospore shower method. Stubble is placed 5 cm above the seedlings and moistened three times daily to allow ascospores to discharge and fall on the seedlings below. (c) An example of seedlings that have been inoculated with a population of *L*. *maculans* with no fungicide resistance. Only the seedlings with no fungicide (bare from Fig 1A) were infected. (d) An example of seedlings that have been inoculated with a population of *L*. *maculans* with high fungicide resistance. Seedlings are infected in all punnets.

An established ‘ascospore shower’ method was adapted to screen for *L*. *maculans* isolates with fungicide resistance during plant infection [18]. Trays containing ten individual punnets were sown with cultivar ATR-Stingray; seeds sown in eight punnets were treated with fluquinconazole and seeds sown in two punnets were not (Fig 1). Ten days after sowing, trays were placed in plastic tubs and stubble, matured as described above, from single sites was moistened and then evenly distributed on mesh 5 cm above the plants. Plastic tubs were then sealed with a lid. Stubble was moistened three times per day to achieve maximum ascospore release. After 30 hours, trays were removed from the plastic tubs and placed in the glasshouse to allow development of disease. Isolates were cultured from lesions as described below.

The *in planta* ascospore shower technique was used to screen the 200 stubble samples collected from around Australia in 2015. Each stubble sample was used to inoculate a single tray of punnets and lesion development was assessed at 21 dpi. For each punnet containing seedlings grown from fluquinconazole-treated seed, the number of seedlings that emerged was determined as well as the number of seedlings with at least one lesion. Therefore, for each stubble sample tested, eight replicate punnets (each containing between 8–12 seedlings grown from fluquinconazole-treated seed), were used to determine the average number of seedlings with at least one lesion. These data were then used to calculate the percentage of diseased seedlings. This percentage was in turn used to determine fungicide-resistant populations. Populations were considered to have no resistance if less than 5% of seedlings were infected, low resistance if 6–20% of seedlings were infected and high resistance if greater than 21% of seedlings were infected.

### Isolates used in this study

Isolates were cultured from lesions that developed on seedlings grown from fluquinconazole-treated seed that had been inoculated with ascospores from stubble screened in 2014 (S1 Table). Using a sterile scalpel blade, lesions were cut out and the material was then surface sterilized. Surface sterilizing was performed in a laminar flow hood by washing leaf tissue with 2% bleach for 2 minutes followed by two minute rinses, twice, in sterile water. Leaf material was then dried on sterile filter paper for 10 minutes before being placed on 10% Campbell’s® V8 juice agar medium. 3–5 days later, asexual fruiting bodies were visible on the leaf tissue when visualised under the dissecting microscope. A sterile needle was used to scoop ooze of asexual spores produced in pycnidia from single fruiting bodies (therefore clonal spores), and then plates were inoculated to produce single isolates. Five resulting isolates from fluquinconazole-treated plants were named 14P286, 14P287, 14P289, 14P290 and 14P291.

In addition to the fungicide resistant isolates, isolates well-characterised previously with respect to their pathogenicity on *B*. *napus* cultivars were used for comparison and for crosses. These were isolates D9 and D13 for plant inoculations and *in vitro* assays and isolates D2 to D10, D13, D14, D16, D17 and 15FRG066 for *in vitro* assays [18] (S2 Table).

### Genetic segregation analyses

Isolates D13 and 14P287, of opposite mating type, were crossed and progeny collected from the F_1_ generation as described by Cozijnsen, Popa [19] and segregating progeny collected to test the inheritance of the fungicide resistant phenotype. As the control to demonstrate the independent assortment of genetic markers in the cross, the mating type locus was used, with the two idiomorphs scored using the PCR assay as described by Cozijnsen and Howlett [20]. Progeny were screened for resistance to fluquinconazole as described below.

### Cultivars and fungicide treatment

Three *B*. *napus* cultivars were used: Westar (no resistance genes against *L*. *maculans*), ATR-Stingray (containing the *Rlm3* major resistance gene) and Pioneer SturtTT (also with the *Rlm3* gene) [18]. The resistance to *Rlm3* has been overcome in Australian *L*. *maculans* populations [18, 21], and therefore all isolates are expected to be virulent towards ATR-Stingray and Pioneer SturtTT. Cultivar ATR-Stingray has high levels of quantitative (also known as minor gene and adult plant) resistance whilst cultivar Pioneer SturtTT has moderate levels of quantitative resistance (https://grdc.com.au/GRDC-FS-BlacklegManagementGuide).

Fluquinconazole (Jockey®) was applied to seed at the label-recommendation rate of 20 litres/tonne. To treat seed, 5–10 grams of seeds were placed in a 50 ml tube, the appropriate quantities of fungicide were added and then the tubes were gently agitated until the fungicide was evenly coating the seeds. Flutriafol (Impact® in furrow) and a standard mixture of prothioconazole/tebuconazole (Prosaro®) were applied to seedlings at the recommended rates of 400 ml ha^-1^ and 450 ml ha^-1^, respectively, as described below.

### Inoculations of seedlings using single isolates

Resistance to fluquinconazole *in planta* was determined by inoculating cotyledons or true leaves (when plants were at the 3-leaf growth stage) of *B*. *napus* seedlings grown from fluquinconazole-treated seed. Inoculations were performed as previously described whereby plant tissue was wounded (four punctures with a 26 gauge needle per cotyledon, two per true leaf) and inoculated with conidia (5 x 10^6^ spores ml^-1^ final concentration) of each isolate [22]. To assess fungicide resistance at the seedling stage, eight plants were screened for each isolate-treatment combination. Symptoms were assessed at 17 days post-inoculation (dpi) on a 0 (no darkening around wounds) to 9 (large grey lesions with prolific sporulation) scale as previously described [23]. The first assessment of fungicide resistance in *L*. *maculans* populations involved inoculation of cotyledons only and comparing the response of isolates on seedlings grown from non-treated seed (bare) to those grown from fluquinconazole-treated seed. To determine whether the efficacy of fluquinconazole decreased as the plant matured, seedlings at the third leaf growth stage were inoculated on their cotyledons, first, second and third true leaves. Disease was then assessed on each tissue type at 17 dpi.

To determine whether the fungicide-resistant isolates caused increased crown canker, cotyledons of seedlings were inoculated at 10 days post emergence, then those infected plants were transferred into pots (20 cm in diameter) and grown to maturity. At maturity the plants were cut at the crown and the cross-section was visually inspected for the percentage blackening (0, 10, 20 to 100%) which indicated disease severity, as previously described [15]. Average disease severity scores were determined from 12 mature plants per isolate-treatment combination. Differences in disease severity were determined by one-way analysis of variance after square root transformation of the data using GenStat® 16^th^ Edition. *P* values less than 0.05 were considered statistically significant.

To screen for cross-resistance to other azole fungicides, a modified *in planta* assay was developed. Eight days after sowing, seedlings of cultivar ATR-Stingray were sprayed with either flutriafol (Impact®) or a standard mixture of prothioconazole/tebuconazole (Prosaro®) using a calibrated 12-volt backpack spray unit with a three-nozzle boom, providing even coverage at the appropriate rates. The sprayed trays of seedlings were left to dry for 24 hours before being inoculated with *L*. *maculans*. Fungicide-treated and untreated seedlings were inoculated with *L*. *maculans* isolates, and lesion development scored at 17 dpi as described above.

### *In vitro* assay for fungicide resistance

Mycelial growth of *L*. *maculans* was measured on potato dextrose agar (PDA) amended with a range of concentrations of technical grade fluquinconazole and tebuconazole dissolved in dimethyl sulfoxide (DMSO; 0.008–10 μg ml^-1^, geometric progression ×2). 9-cm Petri dishes containing the fungicide-amended media were inoculated with an inverted mycelium plug (4 mm diameter) cut from the margin of a 7-day-old actively growing colony. Inoculated plates were kept at 16°C in the dark and mycelial growth rate evaluated once the control (no fungicide) reached the edge of the plate [24]. The colony diameter was measured in two perpendicular directions and values recorded in millimetres. Fungicide testing was replicated three times. The concentration of fungicide resulting in 50% reduction in growth (EC_50_) was calculated in Microsoft Excel by linear regression of log_10_-transformed percentage reduction in growth diameter compared to zero fungicide control, against log_10_-transformed concentration of fungicide. The EC_50_ values were analysed by one-way analysis of variance (ANOVA).

### Characterization of *Cyp51* in fungicide resistant isolates

Genomic DNA was extracted from sensitive and resistant isolates, as previously described [25]. The *Cyp51* gene of *L*. *maculans* (accession number CBX97082.1) was previously cloned using a fragment of the *Saccharomyces cerevisiae ERG11* gene to probe a gene library [26]. BLAST searches using the characterised *Cyp51* genes from *Z*. *tritici* (accession number AAF74756.1) and *B*. *graminis* f. sp. *hordei* (accession number CAC85622.1) further confirmed this as the homolog. Using the *L*. *maculans* genome sequence, two primer sets were designed to amplify and sequence the *Cyp51* gene from isolates. Primer set one (Cyp51-1F CTGGGCCCTGGGAAATAAT, Cyp51-1R CGCCCAGGATAACATGAAGT) amplified an 897 bp fragment whilst primer set two (Cyp51-2F TCATCACTCAAGAAACGGAAGA, Cyp51-2R GCAACCCCTACAAAGCTCAC) amplified an 1148 bp fragment. These fragments overlapped by 224 bp. PCR reactions were carried out as follows: 95°C for 2 mins; 35 cycles of 95°C for 30 sec, 59°C for 30 sec, 72°C for 40 sec; and 72°C for 10 min. The amplified products were Sanger-sequenced at the Australian Genome Research Facility, Melbourne, Australia. Sequences were analysed using Geneious version R9.

### *In planta* quantitative PCR analysis

To assess if the regulation of the *Cyp51* gene was different in the resistant isolates when growing within the plant, the expression of the gene was measured in five isolates obtained during this study (14P286, 14P287, 14P289, 14P290, 14P291) and five previously characterised isolates (D5, D8, D9, D13 and D16). Pycnidiospore suspensions were inoculated onto wounded cotyledons of *B*. *napus* cv. Westar, and twelve 5 mm punches around the wound site were collected seven days post-inoculation. The experiment was done in the absence of fungicide due to the fungicide-sensitive isolates not being able to grow in the presence of the fungicide. For comparison, strain D5 was grown in minimal medium with either glucose or galactose as the carbon source [27]. Total RNA was extracted with TRIzol reagent (Invitrogen, Life Technologies). RNA was reverse transcribed with Superscript IV (Invitrogen). The *Actin* and *Cyp51* genes were amplified (primers for *Actin* MAI0247 CCGTGCAGTCTTTCCTTC and MAI0248 TTTTCCATGTCATCCCAG, and for *Cyp51* MAI0234 TGGAGCAGAAGAAGTTTGTC and MAI0235 GGCGAATTTGGAGTCGAAGG), using the SYBR® FAST Universal qPCR kit (KAPA Biosystems) in a CFX95 Real-Time System PCR machine (Bio-Rad). Amplification conditions were 95°C for 10 mins; then 40 cycles of 95°C for 20 sec, 55°C for 20 sec, 72°C for 20 sec. Expression levels were compared against standard curves generated for DNA isolated from the *Actin* and *Cyp51* fragments.

### *In vitro* quantitative PCR analysis

Fluquinconazole resistant isolates 14P287 and 14P289 and sensitive isolates D4, D8 and 15FRG066 were inoculated in 50 ml potato dextrose broth (PDB; BD Difco^TM^) to a concentration of 10^6^ spores ml^-1^ and grown at 20°C, 100 rpm in darkness with four biological replicates per isolate of both treatments and control cultures. Growth curve analysis of these isolates showed similar growth rates from day three (data not shown). At 72 h post-inoculation, when cultures were in the exponential phase of growth, fluquinconazole in DMSO solution was added to the media to a final concentration equivalent to the mean EC_50_ fluquinconazole concentration (0.1 μg ml^−1^). For the control cultures, an equivalent volume of DMSO solvent was added with no fluquinconazole. At 75 h post-inoculation, cultures were harvested, washed with water treated with diethyl pyrocarbonate, and freeze-dried. RNA was extracted with TRIzol reagent and treated with the Turbo DNA-*free*^TM^ kit (Invitrogen). cDNA synthesis was performed with the iScript cDNA Synthesis Kit (BioRad).

*In vitro* qPCR analysis of the *Cyp51* gene was conducted with Platinum SYBR Green master mix (2 μl 10× buffer, 0.6 μl MgCl, 0.5 μl 10mM dNTP, 0.5 μl SYBR Green, 1 μl 10 mM primer set LMCyp1F TGGCTGTTCTTGCTACCGTTG and LMCyp1R AGAACCACTGCCACCAGGAT, 0.05 μl Platinum Taq, 10.35 μl H_2_O) on a BioRad CFX96 qPCR system using *Actin* as the endogenous control. qPCR parameters were initial denaturation at 95°C for 15 min, followed by 40 cycles of 94°C for 15 sec, 57°C for 30 sec, and 72°C for 30 sec. All reactions were carried out with four biological and two technical replicates each. Relative transcript abundances were calculated using the 2^-ΔCT^ method [28]. Statistical analysis was performed with IBM SPSS, relative expression ratios were compared using Mann-Whitney U test with the significance threshold set at 5%.

## Results

### Development of a novel *in planta* assay used to identify *L*. *maculans* isolates with resistance to the fungicide fluquinconazole

**Fig 2:**
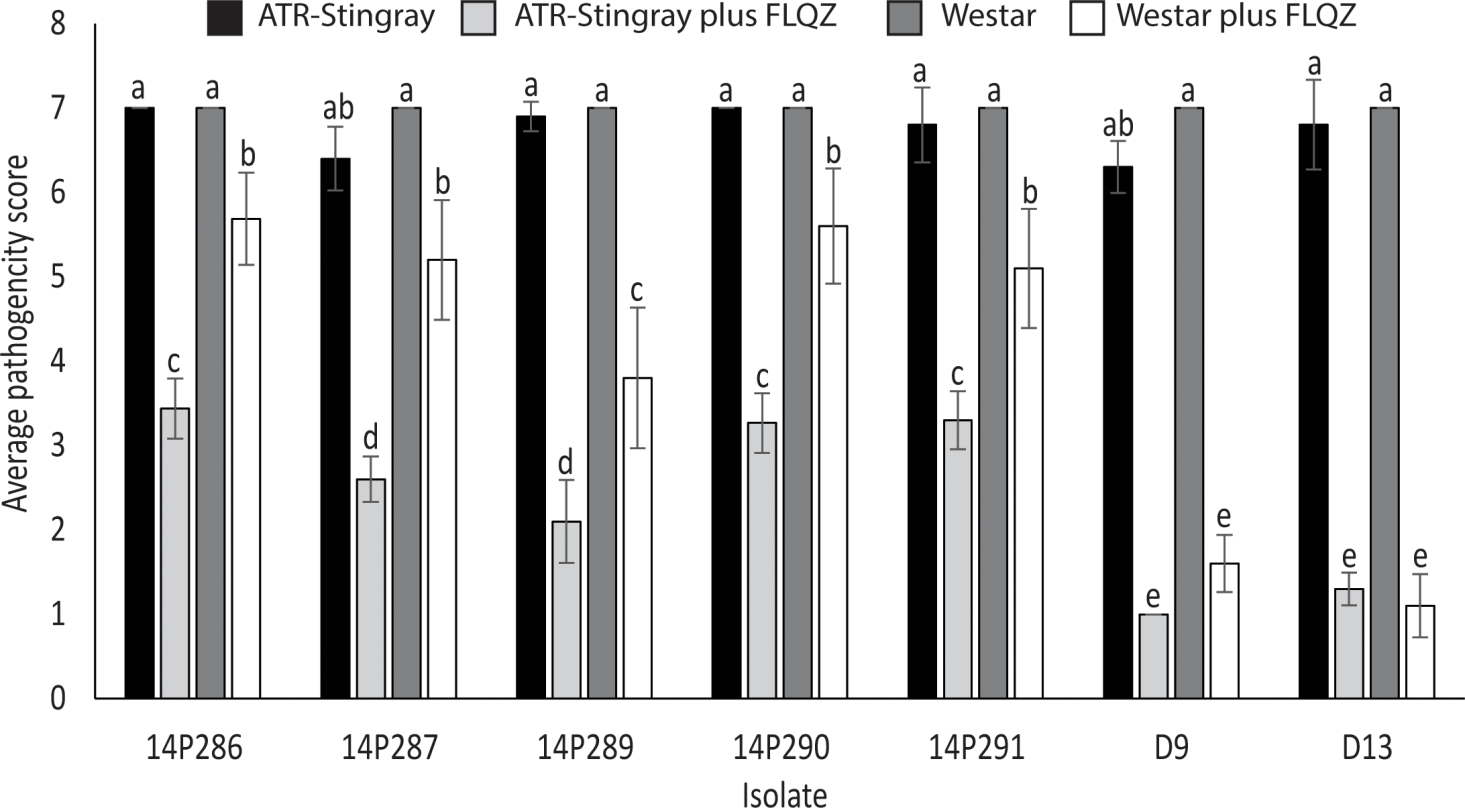
Average disease severity scores of five *Leptosphaeria maculans* fungicide resistant isolates (14P286, 14P287, 14P289, 14P290 and 14P291) compared to sensitive isolates, D9 and D13, when inoculated onto seedlings of cultivars Westar and ATR-Stingray with or without seed treatment with fluquinconazole (FLQZ). Each of the fungicide resistant isolates caused significantly more disease in the presence of fluquinconazole than the sensitive isolates (D9 and D13). Vertical bars represent standard error. The same letter above a bar on the graph indicates a lack of significant difference at p<0.05, as determined by analysis of variation.

Eight stubble samples were collected in 2013 (S1 Table) and used to inoculate seedlings grown from fluquinconazole-treated seed of *B*. *napus* cultivar ATR-Stingray in 2014. Lesions formed on seedlings inoculated with stubble collected from a single site near Katanning, WA. Five isolates (named 14P286, 14P287, 14P289, 14P290 and 14P291) were cultured from lesions from these infected seedlings. When re-inoculated onto either cultivar Westar or ATR-Stingray seedlings grown from fluquinconazole-treated seed, these five isolates produced significantly more disease compared to two well-characterized isolates, D9 and D13 (Fig 2). All isolates were fully virulent on both cultivars Westar and ATR-Stingray in the absence of fungicide. Interestingly, each of the fungicide resistant isolates caused significantly more disease on fluquinconazole-treated seedlings of Westar compared to fluquinconazole-treated seedlings of ATR-Stingray.

### Characterisation of fungicide resistant isolates using *in planta* assays

Since fluquinconazole is applied as a seed dressing, as the plant ages the fungicide becomes more dilute, or degrades, and therefore has less efficacy against *L*. *maculans*. To determine whether the fungicide resistant isolates are more pathogenic compared to control isolates as the fungicide concentration decreases, cotyledons, first, second and third true leaves were inoculated. The fungicide resistant isolates caused significantly more disease than the sensitive isolate (D13) in the presence of fluquinconazole at all growth stages tested (Table 1). In the absence of fungicide, there is significantly less disease present on the 2^nd^ and 3^rd^ true leaves for three of the isolates. This is consistent with a previous report whereby inoculation of true leaves resulted in less disease than the inoculation of the cotyledon counterparts [22]. However, for all five fungicide resistant isolates, there was no significant difference in virulence on fungicide-free versus fluquinconazole-treated seedlings on the first, second and third true leaves (Table 1). For the control isolate, D13, there was significantly less disease on the cotyledons and all true leaves for those plants grown from fungicide treated seed when compared to those grown from seed without treatment.

**Table 1:** Disease severity of fungicide resistant *Leptosphaeria maculans* isolates, 14P286 to 14P291, on different aged leaves in the presence or absence of fluquinconazole compared to the susceptible isolate, D13. All fungicide resistant isolates caused significantly more disease on fluquinconazole-treated seedlings than the sensitive isolate at all growth stages. Although not fully virulent on cotyledons, the fungicide resistant isolates were equally virulent on both bare and fluquinconazole treated seedlings at the 1^st^, 2^nd^ and 3^rd^ leaf stages. Disease severity scores are an average of eight replicate plants.

Isolate	Average disease severity on different leaf types^1^							
	Cotyledons		1st leaf		2nd leaf		3rd leaf	
	Bare	FQNZ^2^	Bare	FQNZ	Bare	FQNZ	Bare	FQNZ
14P286	6.8^a^	2.6^d^	6.3^ab^	6.1^ab^	6.0^ab^	5.1^bc^	6.1^ab^	4.4^bc^
14P287	5.0^bc^	3.3^cd^	5.1^b^	4.5^bc^	4.4^bc^	3.3^cd^	4.5^bc^	4.4^bc^
14P289	4.9^bc^	2.8^cd^	6.1^ab^	4.6^bc^	3.3^cd^	3.5^cd^	3.0^cd^	4.4b^c^
14P290	6.8^a^	2.6^d^	6.0^ab^	5.8^ab^	5.1^bc^	3.8^cd^	3.3^cd^	3.5^cd^
14P291	7.0^a^	3.1^cd^	6.8^a^	5.8^ab^	6.1^ab^	5.5^b^	5.4^b^	5.9^ab^
D13	7.0^a^	1.0^e^	5.1^bc^	1.5^e^	4.1^c^	1.6^e^	3.8^cd^	1.3^e^

^1^ Average disease severity scores followed by the same lower case letter (a through e) are not significantly different at *p* < 0.05, as determined by analysis of variation.

^2^ FQNZ, fluquinconazole.

To determine whether the fungicide resistant isolates cause more disease at the adult growth stage, plants inoculated at the seedling stage (via inoculation of the cotyledons) were grown through to maturity and assessed for severity of stem cankering. The fungicide resistant isolates caused significantly higher disease severity on both cultivar Westar and Pioneer SturtTT compared to the sensitive isolate, D13 (Table 2). No significant differences were observed for fluquinconazole-treated ATR-Stingray: this cultivar is known to have high levels of quantitative resistance, which would result in low levels of disease for all isolates.

**Table 2:** Average disease severity of mature canola plants grown from seedlings inoculated with fungicide resistant (14P286-14P291) or control (D13) *Leptosphaeria maculans* isolates. Seedlings (bare or fluquinconazole-treated) were inoculated with individual isolates and then allowed to grow through to maturity before being assessed for disease severity at the crown of the plant. For cultivars Westar and Pioneer SturtTT, the fungicide resistant isolates cause significantly more disease than the sensitive isolate, D13, in stems of plants grown from fluquinconazole-treated seedlings. No significant differences were observed for ATR-Stingray due to the low levels of disease detected in all isolates. Disease severity was determined as an average of 12 plants.

Cultivar	Isolate	Disease severity at maturity^1^	
		Bare seedlings	fluquinconazole -treated seedlings
Westar	14P286	100^a^	73^b^
	14P287	100^a^	92^a^
	14P289	100^a^	81^ab^
	14P290	100^a^	81^ab^
	14P291	100^a^	59^b^
	D13	100^a^	37^c^
Pioneer SturtTT	14P286	100^a^	52^bc^
	14P287	93^ab^	30^c^
	14P289	90^ab^	66^b^
	14P290	100^a^	61^b^
	14P291	100^a^	16^cd^
	D13	91^ab^	5^d^
ATR-Stingray	14P286	12^b^	3^bc^
	14P287	14^ab^	2^bc^
	14P289	12^b^	3^bc^
	14P290	29^a^	3^bc^
	14P291	8^bc^	2^bc^
	D13	3^bc^	0^c^

^1^ Average disease severity scores followed by the same lower case letter (a through d) for each cultivar are not significantly different at *p* < 0.05, as determined by analysis of variation.

Crosses were set up between isolates D13 (fungicide sensitive isolate, *MAT1-2*) and 14P287 (fungicide resistant isolate, *MAT1-1*), 22 progeny were isolated, and then screened for fluquinconazole resistance using the *in planta* assay. All progeny were screened on two different cultivars (Westar and ATR-Stingray) raised from seed with or without fluquinconazole dressing (Table 3). Of the 22 progeny, ten showed resistance towards fluquinconazole whilst 12 did not. Chi-square analysis shows that this is not significantly different from a 1:1 ratio (0.5<p<0.3). In addition, an exact test of goodness-of-fit indicated that this was not significantly different from a 1:1 ration (*p* = 0.832, two-sided). These data are consistent with the segregation of a single gene, however, a larger number of progeny need to be analysed to provide additional support for this observation.

**Table 3:** Phenotypes in response to fluquinconazole of progeny of a cross between isolates 14P297 and D13.

Isolate	Cultivar and fungicide treatment				Fluquinconazole phenotype
	Westar		ATR-Stingray		
	Bare	Jockey	Bare	Jockey	
14P297	7.0	5.9	7.0	3.4	R
D13	7.0	1.6	7.0	1.1	S
46R01	7.0	3.8	6.6	3.1	R
46R02	6.2	1.3	6.5	1.0	S
46R03	6.3	1.6	5.6	1.7	S
46R04	6.5	3.8	5.9	3.8	R
46R05	7.0	3.7	6.9	3.4	R
46R06	7.0	1.3	7.0	1.1	S
46R07	7.0	1.4	6.8	1.1	S
46R08	7.0	4.3	5.3	3.3	R
46R09	7.0	3.9	5.3	3.5	R
46R10	6.4	1.3	6.0	1.0	S
46R12	7.0	1.0	6.6	1.3	S
46R14	7.0	4.0	7.0	3.8	R
46R16	7.0	6.7	6.6	3.9	R
46R18	7.0	1.4	7.0	1.0	S
46R19	7.0	1.1	7.0	1.1	S
46R20	6.4	3.8	6.9	3.1	R
46R22	6.8	1.4	6.4	1.4	S
46R23	6.9	1.5	6.6	1.4	S
46R25	7.0	5.6	7.0	3.5	R
46R26	7.0	1.4	6.4	1.3	S
46R28	6.1	3.6	5.7	3.4	R
46R29	5.8	1.2	6.6	1.1	S

**Fig 3:**
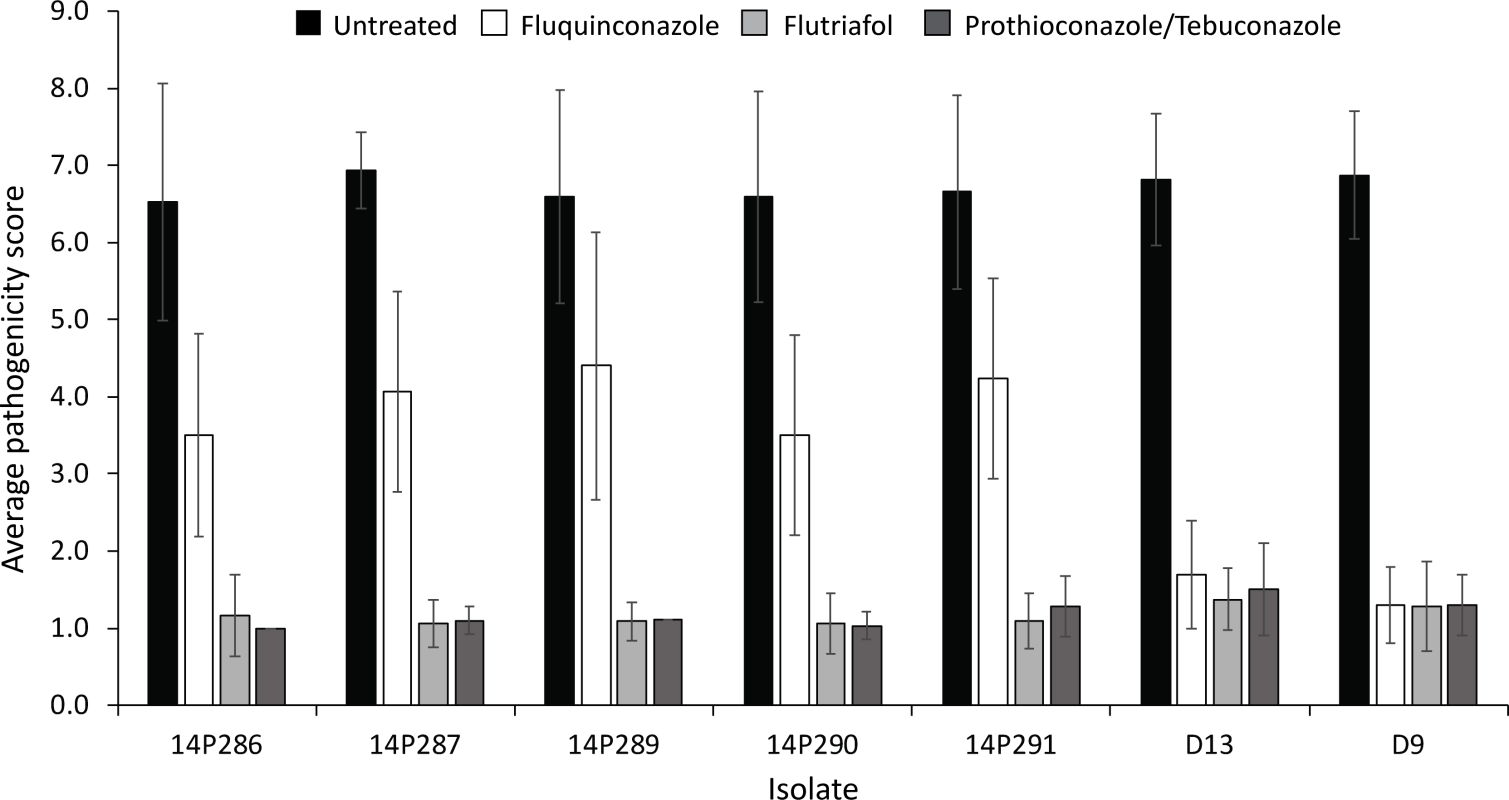
Average disease severity scores of seven *Leptosphaeria maculans* isolates screened on cultivar ATR-Stingray treated with azole fungicides. The five isolates with increased resistance to fluquinconazole (14P286, 14P287, 14P289, 14P290 and 14P291) compared to the sensitive isolates, D13 and D9, showed no increased resistance to flutriafol or the prothioconazole/tebuconazole mixture. Vertical bars represent the standard error of the average scores determined from eight replicate plants.

The fluquinconazole-resistant isolates were tested for possible cross-resistance towards two other azole formulations, flutriafol and the combination prothioconazole/tebuconazole, using the *in planta* assay. As expected, all isolates were virulent on the untreated control. The five fungicide resistant isolates, 14P286-14P291, showed increased resistance on the seedlings grown from seed treated with fluquinconazole, whilst the sensitive isolates, D13 and D9, did not (Fig 3). However, no isolates were resistant towards flutriafol or prothioconazole/tebuconazole, suggesting no cross-resistance occurs between the different azole fungicides.

### Characterisation of fungicide resistant isolates using *in vitro* and molecular assays

**Fig 4:**
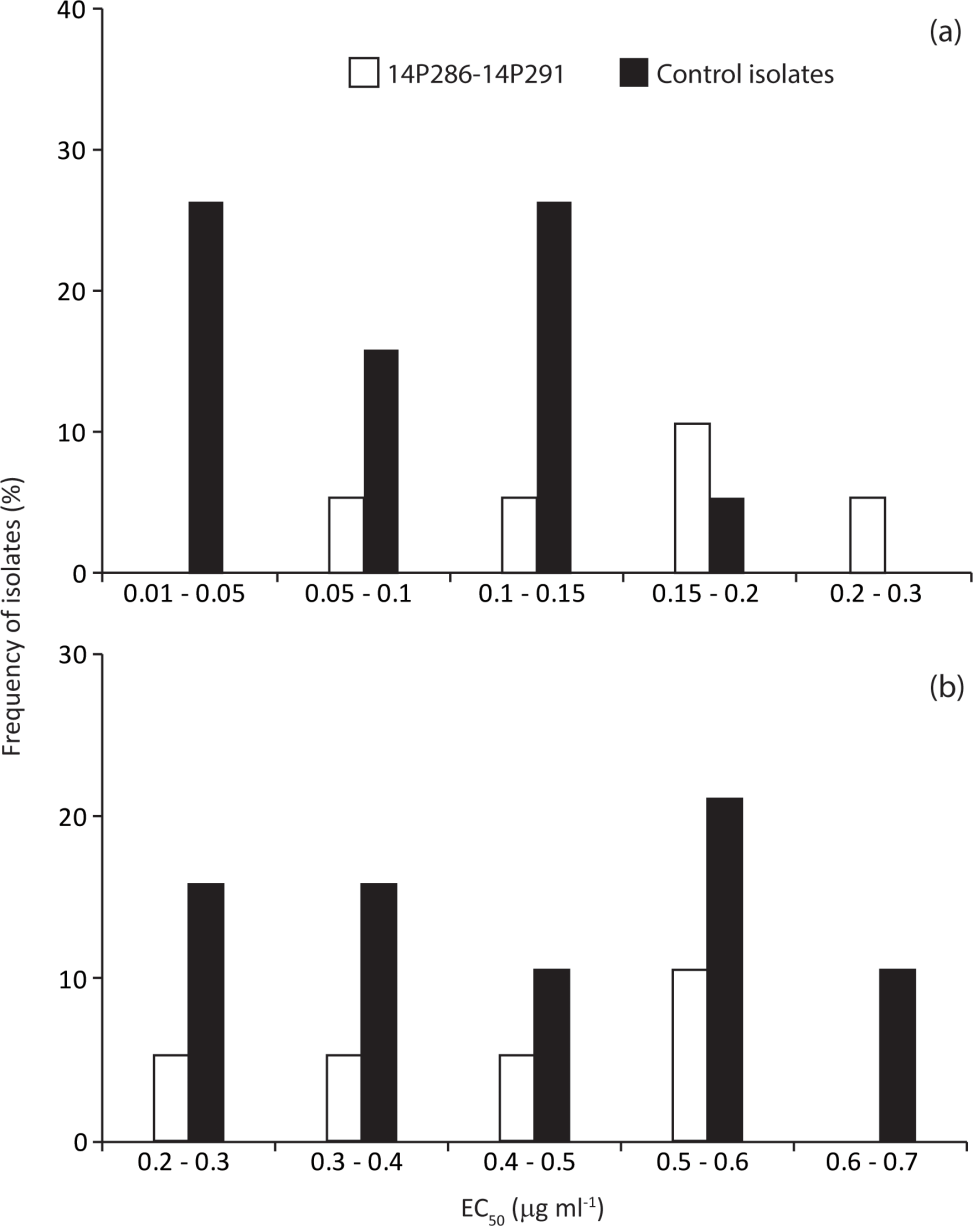
**Frequencies of sensitivity to the DMI fungicides (a) fluquinconazole and (b) tebuconazole in 19 isolates of *Leptosphaeria maculans* including 5 fungicide resistant isolates 14P286 to 14P291 and a subset of 14 sensitive isolates made of 13 previously characterized isolates D2-D10, D13, D14, D16 and D17 and the fungicide sensitivity control isolate 15FRG066.** The radial growth of mycelium on PDA medium amended with technical grade fungicide was measured, and the effective dose at which 50% of growth was inhibited (EC_50_ value) was calculated for each isolate. Dark and light grey bars indicate sensitivity frequencies of resistant and sensitive isolates, respectively.

In addition to the *in planta* assays, *in vitro* assays evaluated the fungicide sensitivity of the five resistant isolates (14P286, 14P287, 14P289, 14P290, 14P291) to determine EC_50_ values to fluquinconazole and tebuconazole fungicides. In addition to the fungicide resistant isolates a set of isolates (D2 to D10, D13, D14, D16 and D17), previously characterized for their avirulence gene profile, and the fungicide sensitive isolate 15FRG066, were used as a wild-type group in the *in vitro* assays. EC_50_ values for *L*. *maculans* wild-type and resistant isolates ranged from 0.01 to 0.2 μg ml^−1^ (average of 0.08 μg ml^−1^) and from 0.06 to 0.21 μg ml^−1^ (average of 0.15 μg ml^−1^), respectively, for fluquinconazole (Fig 4A). However, EC_50_ values for *L*. *maculans* wild-type and resistant isolates ranged from 0.21 to 0.7 μg ml^−1^ (average of 0.41 μg ml^−1^) and from 0.2 to 0.57 μg ml^−1^ (average of 0.44 μg ml^−1^), respectively, for tebuconazole (Fig 4B). For fluquinconazole, the average EC_50_ value of the five *in planta* fluquinconazole-resistant isolates was significantly different to the wild-types (*p* < 0.05). No significant differences between resistant and wild-type isolates were found for tebuconazole (*p* = 0.7).

**Fig 5:**
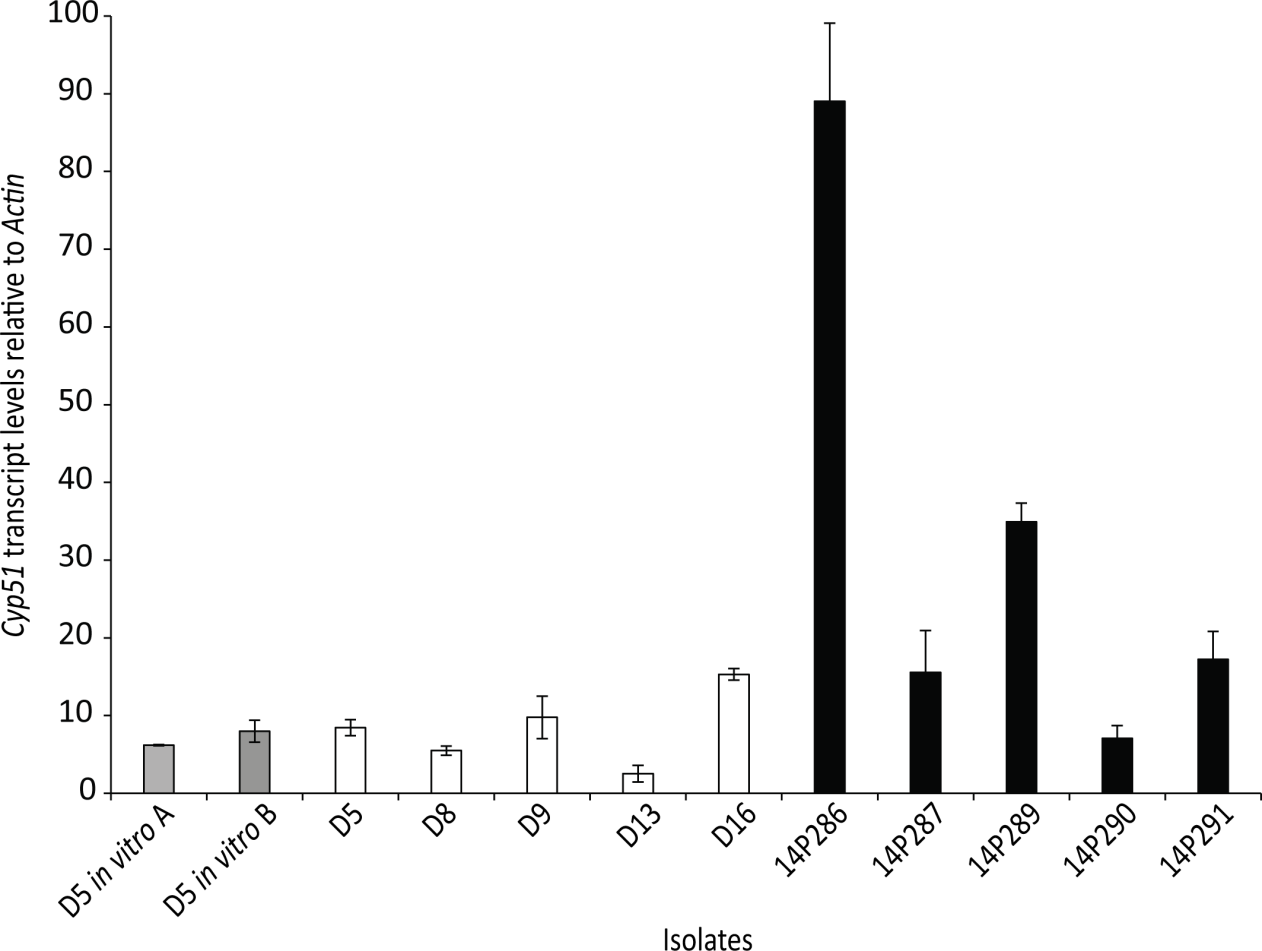
*In planta* expression analysis of the *Cyp51* gene relative to the house-keeping *Actin* gene in 10 isolates of *Leptosphaeria maculans*. **Isolates were inoculated onto *B*. *napus* cv. Westar plants, and tissue harvested 7 dpi.** D5-D16 are previously characterized isolates (white bars) and the five 14P286-14P291 isolates are from plants treated with fluquinconazole (black bars). Strain D5 was also grown *in vitro* in minimal medium (grey bars) with glucose (*in vitro* A) or galactose (*in vitro* B) as the carbon source. Gene expression was measured using qPCR, with error bars indicating standard error from three technical replicates.

Since resistance to triazoles, such as fluquinconazole, is often conferred by changes to the sequence of the target protein, Cyp51, or expression of this gene, sequencing and quantitative RT-PCR were carried out. The *Cyp51* gene was sequenced from 13 susceptible isolates (D2 to D10, D13, D14, D16, D17) and the five fungicide resistant isolates (14P286, 14P287, 14P289, 14P290, 14P291). No mutations were detected throughout the upstream, coding and downstream regions of the *Cyp51* gene for any of the isolates screened (data not shown). Quantitative RT-PCR was then used to look at expression of *Cyp51* in isolates *in planta* at 7 dpi in 10 isolates, including the five fungicide resistant isolates. While variations in expression levels were observed, there was overlapping expression levels between the five susceptible and five resistant isolates (Fig 5).

**Fig 6:**
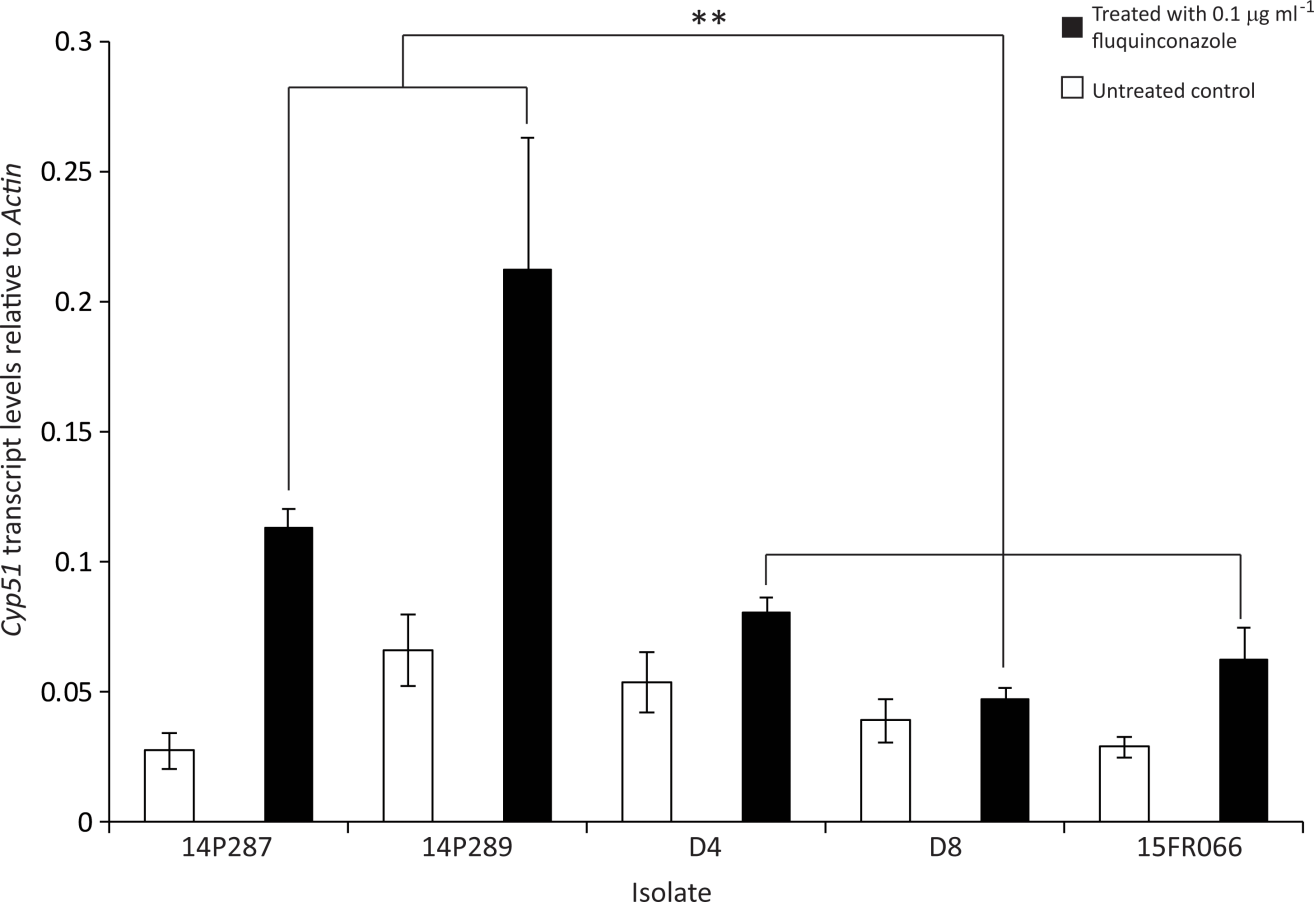
*In vitro* expression analysis of the *Cyp51* gene in fluquinconazole-resistant (14P287 and 14P289) and fluquinconazole-sensitive (D4, D8 and 15FRG066) *Leptosphaeria maculans* isolates following the addition of fluquinconazole (0.1 μg ml^−1^) to cultures. Black and white bars indicate treated (addition of fluquinconazole (0.1 μg ml^−1^)) and untreated cultures, respectively. Values are mean of two biological and four technical replicate experiments with standard deviations shown. The treated cultures of fluquinconazole-resistant isolates (14P287 and 14P289) were significantly different (indicated by asterisk) to those of the of the fluquinconazole-sensitive isolates (D4, D8 and 15FRG066) at *p* < 0.05.

To further investigate the role of *Cyp51* expression on resistance, qPCR analysis of sensitive and resistant isolates induced *in vitro* with the population mean EC_50_ fluquinconazole concentration (0.1 μg mL^−1^) was undertaken. Analysis comparing three fluquinconazole-sensitive (D4, D8 and 15FRG66) and two resistant (14P287 and 14P289) isolates revealed the average expression of *Cyp51* to be 2.6-fold higher in the resistant compared to the sensitive isolates, which was considered significant (Mann–Whitney *U* = 9, *p* < 0.0001) (Fig 6). By contrast, the average *Cyp51* expression levels were not significantly different in the DMSO-treated samples of both resistant and sensitive isolates (Mann–Whitney *U* = 157 *p* = 0.345) (Fig 6).

### Estimating the proportion of fungicide resistance in *L*. *maculans* populations across Australia

**Fig 7:**
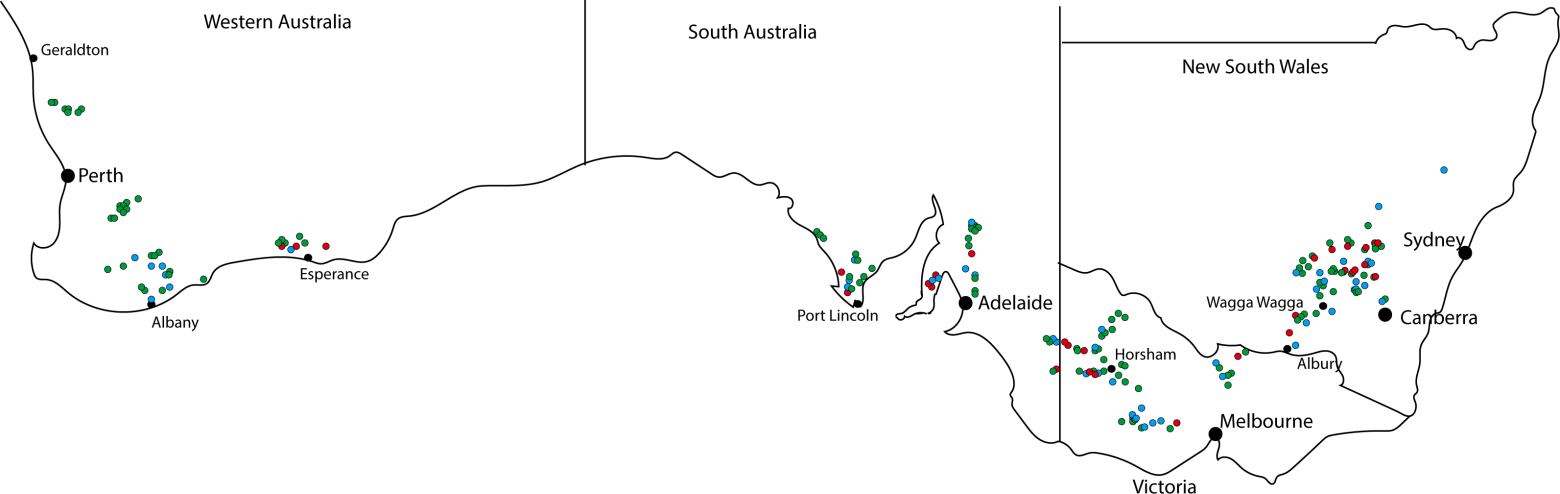
Survey for fungicide resistance in 200 *Leptosphaeria maculans* populations from across Australia. Red dots indicate populations from which isolates were obtained that were able to cause high levels of disease on plants arising from fungicide-treated seed, blue dots indicate populations with low levels of resistance, and green dots indicate populations from which no isolates were obtained that had resistance.

To determine how wide spread the fungicide resistance is in Australian populations, a survey of stubble collected from 200 paddocks dispersed across the Australian canola growing regions (Fig 7) was undertaken. From these 200 samples, 15% contained resistant *L*. *maculans* ascospores that infected the cotyledons treated with fluquinconazole, with the percentage across the states varying from 7% in Western Australia to 20% in New South Wales (Table 4). There was no correlation between region, prior history of fungicide application and/or variety with fungicide resistance (S1 Table).

**Table 4:** Frequency of fungicide resistance in Australian *Leptosphaeria maculans* populations.

State	Number of samples	Percentage of population (number of isolates)^a^		
		No resistance	Low resistance	Resistant
VIC	50	60 (30)	26 (13)	14 (7)
SA	42	60 (25)	23 (10)	17 (7)
NSW	66	54 (36)	26 (17)	20 (13)
WA	42	76 (32)	17 (7)	7 (3)
Total	200	62 (123)	23 (47)	15 (30)

^a^ Populations were considered to have no resistance if less than 5% of cotyledons were infected, low resistance if 6–20% of cotyledons were infected and high resistance if greater than 21% of cotyledons were infected.

## Discussion and conclusion

Fungicides are an integral part of the control of plant pathogens. The use of fungicides to control blackleg disease of canola is essential in maintaining current production levels in many countries, and therefore understanding the fungal evolution to these fungicides is vital to be able to continue using these chemicals. In the current study, we developed a high-throughput screen for detecting strains able to cause disease on plants raised from seed treated with a triazole fungicide. This method screens several thousands of isolates per population as the spores are released from the stubble, and as such can detect within a population a small minority of isolates that can potentially replace the current population, in a manner similar to that shown for population replacement in response to selection imposed by particular resistances genes in canola cultivars [29, 30]. This assay has several advantages. First, in contrast to *in vitro* assays whereby only small numbers of isolates are generally screened at any one time, the *in planta* assay screens several thousands of spores, each representing a single isolate. Second, it is of greater physiological relevance to what occurs in the field than *in vitro* assays. This type of *in planta* assay can potentially be used to screen for fungicide resistance in populations of any stubble-borne pathogen.

Five fungicide resistant isolates from stubble collected in 2013 were identified and characterised further. This is the first time altered responses to a triazole fungicide have been detected in *L*. *maculans*. Although these isolates caused significantly more disease than the susceptible controls when inoculated onto seedlings grown from fluquinconazole-treated seed, this increase in resistance was only observed as a slight shift in sensitivity *in vitro*. It is worthy to note that the shift in average EC_50_ using *in vitro* assays would not have been detected if the fungicide resistant isolates had not already been characterised using the *in planta* assay. Unfortunately, very few studies have been published that investigated fungicide sensitivity in *L*. *maculans* using *in vitro* assays and those that have, have focused on strobilurins [31, 32]. Minimal correlation between *in vitro* and *in planta* assays have also been reported for *Phaeosphaeria nodorum* with selection assays for resistance to a strobilurin, azoxystrobin [33]. Using an *in planta* selection assay, increases in tolerance to azoxystrobin were detected after four generations; however, this was not detected in the *in vitro* assays [33]. One possible reason for the inconsistency between the *in planta* and *in vitro* assays in the current study may be that the gene(s) conferring the increased resistance are only expressed *in planta* or require some sort of induction. The latter option is consistent with the finding that *Cyp51* was more highly expressed in the fungicide resistant isolates compared to the sensitive isolates, following spiking of the *in vitro* cultures with fluquinconazole.

Interestingly, the fluquinconazole fungicide was more effective when applied to cultivars ATR-Stingray and Pioneer SturtTT than on the highly susceptible cultivar Westar. This was the case at both the seedling stage (less disease on cotyledons of the fungicide treated seedlings than the bare controls) and maturity (less internal infection in the fungicide treated plants compared to the bare controls). This interaction between host genotype and fungicide efficacy is consistent with reports in other diseases such as Fusarium Wilt in Chickpea and Downy Mildew in Cucumber, whereby fungicide efficacy differs in the presence of host resistance [34, 35]. However, the reasons for this interaction remain unknown. In the situation reported in the current study, both ATR-Stingray and Pioneer SturtTT harbour resistance gene, *Rlm3*. It would be worth investigating whether similar interactions occur between other major resistance genes and other fungicides.

No consistent expression differences were observed for *Cyp51 in planta* between the fungicide resistant and sensitive isolates. In addition, no mutations were detected within the coding or promoter regions of the *Cyp51* gene. However, expression of *Cyp51 in vitro* after induction with fluquinconazole revealed significant differences when average expression levels of the resistant and sensitive isolates tested were compared. One possible explanation for this result is that the resistant isolates might respond to the presence of fungicide more rapidly than sensitive isolates. Although the molecular mechanism behind this is currently unknown, segregation of progeny suggests that a single gene is involved. However, since only 22 progeny were analysed in this study, further analyses of the genetic heritability of this trait is required to confirm the segregation of a single gene. The identity of the genetic basis for resistance is currently being sought using a number of molecular biology methods.

In the Australian canola industry, prior to 2017 all registered fungicides for use against blackleg belong to the triazole class; however, we have shown that there is no cross resistance between fluquinconazole and two other fungicides options used to combat blackleg. This is consistent with other species such as isolates of *Z*. *tritici* that are highly resistant to tebuconazole but susceptible to a second triazole, prochloraz [36].

In the survey across Australia, 15% of stubble samples were identified as containing isolates able to cause disease on seedlings that had been treated with fluquinconazole. It is unknown whether this frequency is increasing, decreasing or remaining constant since this was the first time such as survey was carried out. Fluquinconazole has been used in Australia routinely since the mid-2000s [16] and therefore there has been constant selection pressure on the population for resistance to increase in frequency. Furthermore, the current study found that these fungicide resistant isolates could cause significantly more disease in the stems of mature plants that had grown from seed dressed with fluquinconazole, the tissue of the plant where the fungus colonizes and undergoes sexual reproduction in the following season. Therefore, one would expect that these isolates will increase in frequency the following season. Regardless of whether the frequency of these isolates is increasing in the population, this study shows that fungicide use needs to be managed in Australian farming systems and suggests that in regions where resistance to fluquinconazole has been detected, farmers may need to rely more heavily on other control strategies such as growing cultivars with high levels of resistance to blackleg, rotation of resistance genes and avoidance of last year’s stubble [13, 15, 18].

## Supporting information

S1 Table Details of stubbles used in this study. (DOCX)

S2 Table Fungicide sensitive isolates used in this study. (DOCX)
