# Supplementary material for: Identification of isolates of the plant pathogen Leptosphaeria maculans with resistance to the triazole fungicide fluquinconazole using a novel In Planta assay
Source: PLoS One. 2017 Nov 15;12(11):e0188106. doi: 10.1371/journal.pone.0188106 (PMC5687775; doi:10.1371/journal.pone.0188106)
Supplement: S1 Table — (DOCX) [file pone.0188106.s001.docx]

**S1 Table. Details of stubbles used in this study.**

| **Year**  **sown** | **State** | **Closest town** | **Variety** | **Fungicide details** | **Tolerance status** |
| --- | --- | --- | --- | --- | --- |
| 2014 | SA | Wanilla | Hyola444TT | Jockey | No tolerance |
| 2014 | Vic | Wonwondah | 45Y86CL | Jockey | No tolerance |
| 2014 | Vic | Wonwondah | 45Y86CL | Prosaro @ 375ml/ha @ 4-6 leaf stage | No tolerance |
| 2014 | Vic | Wonwondah | 45Y86CL | None | No tolerance |
| 2014 | Vic | Wonwondah | 45Y86CL | Jockey, Prosaro @ 375ml/ha @ 4-6 leaf stage | No tolerance |
| 2014 | WA | Katanning | CB Atomic | Impact in Furrow | No tolerance |
| 2014 | WA | Katanning | CB Atomic | Jockey | No tolerance |
| 2014 | WA | Katanning | CB Atomic | Prosaro @ 375ml/ha @ 4-6 leaf stage | Tolerance |
| 2015 | NSW | Ardlethan | AVGARNET | Jockey | Low tolerance |
| 2015 | NSW | Ardlethan | ATRGEM | Jockey | No tolerance |
| 2015 | NSW | Ardlethan | ATRBONITO | Jockey | No tolerance |
| 2015 | NSW | Ardlethan | ATRGEM | Jockey | No tolerance |
| 2015 | NSW | Ariah Park | CRUSHERTT | Impact in Furrow | Low tolerance |
| 2015 | NSW | Ariah Park | ATRSTINGRAY | Jockey, Impact in Furrow | No tolerance |
| 2015 | NSW | Ariah Park | ATRGEM | Jockey, Impact in Furrow | Tolerance |
| 2015 | NSW | Beckom | CRUSHERTT | None | No tolerance |
| 2015 | NSW | Bribbaree | ATRGEM | Jockey, Impact in Furrow | Low tolerance |
| 2015 | NSW | Caragabal | ATRGEM | Jockey | No tolerance |
| 2015 | NSW | Coolamon | ATRGEM | Maxim, Impact in Furrow | No tolerance |
| 2015 | NSW | Coolamon | ATRGEM | Jockey, Impact in Furrow | No tolerance |
| 2015 | NSW | Coolamon | ATRGEM | Jockey, Impact in Furrow | Low tolerance |
| 2015 | NSW | Coolamon | HYOLA559TT | Jockey, Impact in Furrow | No tolerance |
| 2015 | NSW | Coolamon | ATRSTINGRAY | Maxim, Impact in Furrow | No tolerance |
| 2015 | NSW | Coolamon | ATRGEM | Jockey, Impact in Furrow | Low tolerance |
| 2015 | NSW | Cootamundra | CRUSHERTT | None | Low tolerance |
| 2015 | NSW | Cowra | PIONEER44Y87CL | Jockey, Impact in Furrow, Prosaro @ 375ml/ha @ 4-6 leaf stage | No tolerance |
| 2015 | NSW | Cowra | PIONEER45Y86CL | Jockey, Impact in Furrow | No tolerance |
| 2015 | NSW | Cowra | PIONEER45Y86CL | Jockey, Impact in Furrow | Tolerance |
| 2015 | NSW | Cowra | ATRGEM | Jockey, Impact in Furrow | No tolerance |
| 2015 | NSW | Cowra | CRUSHERTT | Jockey | Tolerance |
| 2015 | NSW | Cowra | ATRGEM | Jockey, Impact in Furrow | No tolerance |
| 2015 | NSW | Cowra | ATRGEM | Jockey | Low tolerance |
| 2015 | NSW | Goulburn | CRUSHERTT | Jockey, Impact in Furrow, Prosaro @ 375ml/ha @ 4-6 leaf stage, Prosaro post grazing | No tolerance |
| 2015 | NSW | Goulburn | CRUSHERTT | Jockey, Impact in Furrow, Prosaro @ 375ml/ha @ 4-6 leaf stage, Prosaro post grazing | Low tolerance |
| 2015 | NSW | Greenethorpe | ATRGEM | Jockey, Impact in Furrow | No tolerance |
| 2015 | NSW | Grenfell | ATRSTINGRAY | None | No tolerance |
| 2015 | NSW | Grenfell | CRUSHERTT | Jockey | Tolerance |
| 2015 | NSW | Grenfell | ATRGEM | Jockey | Low tolerance |
| 2015 | NSW | Grenfell | ATRGEM | Jockey | No tolerance |
| 2015 | NSW | Harden | HYOLA559TT | Maxim, Impact in Furrow | No tolerance |
| 2015 | NSW | Harden | AVGARNET | Jockey, Impact in Furrow, Prosaro @ 450 ml/ha post 4-6 leaf stage | Tolerance |
| 2015 | NSW | Harden | SENSATION | Jockey, Impact in Furrow | No tolerance |
| 2015 | NSW | Iandara | ATRGEM | Jockey | Tolerance |
| 2015 | NSW | Iandara | ATRGEM | Jockey, Prosaro @ 450ml/ha @ 4-6 leaf stage | Tolerance |
| 2015 | NSW | Jugiong | HYOLA559TT | Jockey, Impact in Furrow | Low tolerance |
| 2015 | NSW | Junee | PIONEER45Y88CL | Jockey, Impact in Furrow | No tolerance |
| 2015 | NSW | Ladysmith | ATRGEM | Jockey, Impact in Furrow | Low tolerance |
| 2015 | NSW | Lockhart | CRUSHERTT | None | Tolerance |
| 2015 | NSW | Lockhart | PIONEER45Y86CL | Jockey, Impact in Furrow | No tolerance |
| 2015 | NSW | Lockhart | ATRSTINGRAY | Jockey, Impact in Furrow | Low tolerance |
| 2015 | NSW | Lockhart | ATRGEM | Jockey, Impact in Furrow | No tolerance |
| 2015 | NSW | Osborne | ATRGEM | Jockey, Impact in Furrow | No tolerance |
| 2015 | NSW | Parkes | CRUSHERTT | None | No tolerance |
| 2015 | NSW | Quandialla | AVGARNET | Jockey | Tolerance |
| 2015 | NSW | Springdole | PIONEER44Y84CL | Jockey, Impact in Furrow, Prosaro @ 450 ml/ha post 4-6 leaf stage | No tolerance |
| 2015 | NSW | Stockinbingal | PIONEER45Y86CL | Jockey, Impact in Furrow, Prosaro @ 450 ml/ha post 4-6 leaf stage | No tolerance |
| 2015 | NSW | Tamworth | CRUSHERTT | None | Low tolerance |
| 2015 | NSW | Temora | ATRGEM | Jockey, Impact in Furrow | No tolerance |
| 2015 | NSW | Temora | PIONEER44Y84CL | Jockey, Impact in Furrow, Prosaro @ 450 ml/ha post 4-6 leaf stage | Tolerance |
| 2015 | NSW | Temora | UNKNOWN | Jockey, Impact in Furrow | Tolerance |
| 2015 | NSW | Temora | CRUSHERTT | Jockey, Impact in Furrow | No tolerance |
| 2015 | NSW | The Rock | ATRGEM | Jockey, Impact in Furrow | No tolerance |
| 2015 | NSW | Wagga | T28156 | None | Low tolerance |
| 2015 | NSW | Walbundrie | HYOLA404RR | Jockey, Impact in Furrow, Prosaro @ 450 ml/ha post 4-6 leaf stage | Tolerance |
| 2015 | NSW | Wallendbeen | HYOLA577CL | Maxim, Impact in Furrow | No tolerance |
| 2015 | NSW | Wellington | HYOLA450TT | Jockey | Low tolerance |
| 2015 | NSW | Yerong Creek | ATRGEM | Jockey, Impact in Furrow | No tolerance |
| 2015 | NSW | Young | HYOLA559TT | Jockey | No tolerance |
| 2015 | NSW | Young | ATRGEM | Jockey | Tolerance |
| 2015 | NSW | Young | ATRGEM | Jockey | Low tolerance |
| 2015 | NSW | Young | HYOLA559TT | Jockey | Low tolerance |
| 2015 | NSW | Young | HYOLA559TT | Jockey, Impact in Furrow | No tolerance |
| 2015 | NSW | Young | ATRWAHOO | UNKNOWN | No tolerance |
| 2015 | NSW | Young | ATRGEM | Jockey, Impact in Furrow | No tolerance |
| 2015 | SA | Arthurton | UNKNOWN | UNKNOWN | Low tolerance |
| 2015 | SA | Bordertown | CRUSHERTT | Impact in Furrow | No tolerance |
| 2015 | SA | Bordertown | ATRMARLIN | None | Low tolerance |
| 2015 | SA | Bordertown | UNKNOWN | UNKNOWN | Low tolerance |
| 2015 | SA | Clare | AVGARNET | Jockey | Tolerance |
| 2015 | SA | Clinton Centre | HYOLA555TT | Jockey | Tolerance |
| 2015 | SA | Cummins | AVZIRCON | Jockey, Impact in Furrow | No tolerance |
| 2015 | SA | Cummins | PIONEER44Y87CL | Jockey, Impact in Furrow | No tolerance |
| 2015 | SA | Edillie | HYOLA575CL | Jockey, Impact in Furrow | Low tolerance |
| 2015 | SA | Eyre Peninsula | ATRSTINGRAY | Jockey, Prosaro @ 450ml/ha @ 4-6 leaf stage | Low tolerance |
| 2015 | SA | Eyre Peninsula | CBTELFER | Jockey, Prosaro @ 450ml/ha @ 4-6 leaf stage | Low tolerance |
| 2015 | SA | Frances | UNKNOWN | UNKNOWN | No tolerance |
| 2015 | SA | Frances | UNKNOWN | UNKNOWN | Tolerance |
| 2015 | SA | Freeling | HYOLA555TT | Jockey, Impact in Furrow | No tolerance |
| 2015 | SA | Hoyleton | HYOLA559TT | Jockey | Low tolerance |
| 2015 | SA | Jamestown | HYOLA559TT | Jockey | No tolerance |
| 2015 | SA | Jamestown | ATRBONITO | Jockey | Low tolerance |
| 2015 | SA | Jamestown | ATRBONITO | UNKNOWN | No tolerance |
| 2015 | SA | Jamestown | ATRBONITO | Jockey | No tolerance |
| 2015 | SA | Jamestown | HYOLA559TT | Jockey, Impact in Furrow | No tolerance |
| 2015 | SA | Jamestown | CRUSHERTT | Jockey | No tolerance |
| 2015 | SA | Jamestown | CRUSHERTT | Jockey, Impact in Furrow | No tolerance |
| 2015 | SA | Kapinnie | PIONEER45Y86CL | Jockey, Impact in Furrow | No tolerance |
| 2015 | SA | Karkoo | PIONEER44Y87CL | Jockey, Impact in Furrow | No tolerance |
| 2015 | SA | Klangary | UNKNOWN | Jockey, Impact in Furrow | No tolerance |
| 2015 | SA | Lock | ATRSTINGRAY | Jockey | Low tolerance |
| 2015 | SA | Maitland | AVZIRCON | Jockey | Tolerance |
| 2015 | SA | Maitland | AVGARNET | Jockey | Tolerance |
| 2015 | SA | Mt Hope | CBTELFER | None | Tolerance |
| 2015 | SA | Mundulla | AVGARNET | Jockey | No tolerance |
| 2015 | SA | Pt Kenny | ATRSTINGRAY | Jockey | No tolerance |
| 2015 | SA | Pt Kenny | ATRSTINGRAY | Jockey, Impact in Furrow | No tolerance |
| 2015 | SA | Riverton | ATRMARLIN | None | Low tolerance |
| 2015 | SA | Spalding | CBTELFER | None | No tolerance |
| 2015 | SA | Stokes | AVZIRCON | Jockey, Impact in Furrow | No tolerance |
| 2015 | SA | Streaky Bay | ATRSTINGRAY | Jockey, Impact in Furrow | No tolerance |
| 2015 | SA | Tarlee | HYOLA555TT | Jockey | No tolerance |
| 2015 | SA | Templers | HYOLA575CL | Jockey | No tolerance |
| 2015 | SA | Tumby Bay | PIONEER43C80CL | UNKNOWN | No tolerance |
| 2015 | SA | Tumby Bay | ATRSTINGRAY | Jockey, Impact in Furrow | No tolerance |
| 2015 | SA | Wangary | ATRSTINGRAY | Jockey, Impact in Furrow, Prosaro @ 400ml/ha @ 4-6 leaf stage | Tolerance |
| 2015 | SA | Wangary | HYOLA575CL | Jockey, Impact in Furrow | No tolerance |
| 2015 | Vic | Caniambo | CRUSHERTT | Impact in Furrow, Prosaro @ 450ml/ha post 4-6 leaf stage | No tolerance |
| 2015 | Vic | Cosgrove | ATRGEM | Impact in Furrow, Prosaro @ 450ml/ha post 4-6 leaf stage | No tolerance |
| 2015 | Vic | Cosgrove | HYOLA559TT | Impact in Furrow, Prosaro @ 450ml/ha post 4-6 leaf stage | Low tolerance |
| 2015 | Vic | Diggora | UNKNOWN | UNKNOWN | No tolerance |
| 2015 | Vic | Dookie | ATRGEM | Impact in Furrow, Prosaro @ 450ml/ha post 4-6 leaf stage | No tolerance |
| 2015 | Vic | Dookie | ATRGEM | Impact in Furrow, Prosaro @ 450ml/ha post 4-6 leaf stage | No tolerance |
| 2015 | Vic | Drung | MONOLA314TT | Jockey | No tolerance |
| 2015 | Vic | Glenthompson | UNKNOWN | UNKNOWN | No tolerance |
| 2015 | Vic | Gymbowen | UNKNOWN | UNKNOWN | Tolerance |
| 2015 | Vic | Hopetoun | ATRBONITO | Jockey | No tolerance |
| 2015 | Vic | Hopetoun | ATRSTINGRAY | Jockey, Impact in Furrow | No tolerance |
| 2015 | Vic | Horsham | UNKNOWN | UNKNOWN | No tolerance |
| 2015 | Vic | Kaniva | CBTELFER | None | Tolerance |
| 2015 | Vic | Kaniva | UNKNOWN | UNKNOWN | Tolerance |
| 2015 | Vic | Kaniva | UNKNOWN | UNKNOWN | No tolerance |
| 2015 | Vic | Kaniva | UNKNOWN | UNKNOWN | No tolerance |
| 2015 | Vic | Katamatite | UNKNOWN | UNKNOWN | Tolerance |
| 2015 | Vic | Laharum | UNKNOWN | UNKNOWN | No tolerance |
| 2015 | Vic | Lake Bolac | UNKNOWN | UNKNOWN | Low tolerance |
| 2015 | Vic | Lake Bolac | UNKNOWN | UNKNOWN | No tolerance |
| 2015 | Vic | Lake Bolac | UNKNOWN | UNKNOWN | No tolerance |
| 2015 | Vic | Mitre | CRUSHERTT | Jockey, Impact in Furrow | No tolerance |
| 2015 | Vic | Mitre | UNKNOWN | UNKNOWN | Low tolerance |
| 2015 | Vic | Mt Aralpiies | UNKNOWN | UNKNOWN | Tolerance |
| 2015 | Vic | Mt Drysden | ATRGEM | Jockey | No tolerance |
| 2015 | Vic | Murtoa | ATRSTINGRAY | Impact in Furrow | No tolerance |
| 2015 | Vic | Murtoa | ATRSTINGRAY | Impact in Furrow | No tolerance |
| 2015 | Vic | Natimuk | UNKNOWN | UNKNOWN | Low tolerance |
| 2015 | Vic | Netherby | ATRSTINGRAY | Impact in Furrow | Low tolerance |
| 2015 | Vic | Netherby | ATRSTINGRAY | Jockey, Impact in Furrow | No tolerance |
| 2015 | Vic | Netherby | ATRSTINGRAY | UNKNOWN | No tolerance |
| 2015 | Vic | Nhill | ATRGEM | None | Low tolerance |
| 2015 | Vic | Nhill | ATRGEM | None | No tolerance |
| 2015 | Vic | Nhill | PIONEER43C80CL | Impact in Furrow | No tolerance |
| 2015 | Vic | Pimpinio | CRUSHERTT | None | No tolerance |
| 2015 | Vic | Rainbow | ATRSTINGRAY | Impact in Furrow | No tolerance |
| 2015 | Vic | Rossbridge | UNKNOWN | UNKNOWN | Low tolerance |
| 2015 | Vic | Serviceton | ATRBONITO | Jockey | Tolerance |
| 2015 | Vic | Skipton | ATRWAHOO | Maxim, Jockey, Impact in Furrow | Tolerance |
| 2015 | Vic | Skipton | UNKNOWN | UNKNOWN | No tolerance |
| 2015 | Vic | Streatham | UNKNOWN | UNKNOWN | Low tolerance |
| 2015 | Vic | Wahring | THUMPERTT | Prosaro @ 375ml/ha @ 4-6 leaf stage, Prosaro @ 450ml/ha post 4-6 leaf stage | No tolerance |
| 2015 | Vic | Westmere | UNKNOWN | UNKNOWN | Low tolerance |
| 2015 | Vic | Wickliff | UNKNOWN | UNKNOWN | Low tolerance |
| 2015 | Vic | Wickliff | UNKNOWN | UNKNOWN | No tolerance |
| 2015 | Vic | Wickliff | UNKNOWN | UNKNOWN | Low tolerance |
| 2015 | Vic | Wonwondah | PIONEER45Y86CL | Jockey, Prosaro @ 450ml/ha @ 4-6 leaf stage | Low tolerance |
| 2015 | Vic | Wunghnu | UNKNOWN | UNKNOWN | Low tolerance |
| 2015 | Vic | Yapeet | PIONEER43C80CL | Impact in Furrow | No tolerance |
| 2015 | Vic | Yarrawonga | CRUSHERTT | None | No tolerance |
| 2015 | WA | Albany | PIONEER44Y24RR | Jockey | Low tolerance |
| 2015 | WA | Amelup | CRUSHERTT | Impact in Furrow | Low tolerance |
| 2015 | WA | Badgebup | NUSEEDGT50 | Jockey | No tolerance |
| 2015 | WA | Badgingarra | CBTELFER | None | No tolerance |
| 2015 | WA | Badgingarra | ATRSTINGRAY | Jockey | No tolerance |
| 2015 | WA | Bindi Bindi | ATRSTINGRAY | Impact in Furrow | No tolerance |
| 2015 | WA | Bindi Bindi | NUSEEDGT41 | Impact in Furrow, Prosaro @ 450ml/ha post 4-6 leaf stage | No tolerance |
| 2015 | WA | Borden | ATRSTINGRAY | Jockey | Low tolerance |
| 2015 | WA | Boyup Brook | CRUSHERTT | Jockey, Impact in Furrow | No tolerance |
| 2015 | WA | Boyup Brook | CRUSHERTT | Jockey, Impact in Furrow | No tolerance |
| 2015 | WA | Cascade | THUMPERTT | Impact in Furrow | No tolerance |
| 2015 | WA | Condingup | THUNDERTT | Prosaro @ 400ml/ha @ 4-6 leaf stage | Tolerance |
| 2015 | WA | Coomalbidgup | CRUSHERTT | Impact in Furrow | Low tolerance |
| 2015 | WA | Corrigin | CBTELFER | None | No tolerance |
| 2015 | WA | Dalyup | CRUSHERTT | Jockey | Tolerance |
| 2015 | WA | Gairdner River | THUMPERTT | Impact in Furrow | No tolerance |
| 2015 | WA | Gibson | CRUSHERTT | None | No tolerance |
| 2015 | WA | Gibson | HYOLA505RR | Maxim, Impact in Furrow | No tolerance |
| 2015 | WA | Katanning | CRUSHERTT | None | No tolerance |
| 2015 | WA | Katanning West | ATRSTINGRAY | Impact in Furrow | Low tolerance |
| 2015 | WA | Kendenup | ATRMARLIN | None | No tolerance |
| 2015 | WA | Kojonup | ATRMARLIN | None | No tolerance |
| 2015 | WA | Moora | NUSEEDGT50 | Impact in Furrow, Prosaro @ 450ml/ha post 4-6 leaf stage | No tolerance |
| 2015 | WA | Moora | PIONEER43Y23RR | Impact in Furrow, Prosaro @ 450ml/ha post 4-6 leaf stage | No tolerance |
| 2015 | WA | Moora | ATRGEM | Impact in Furrow, Prosaro @ 450ml/ha post 4-6 leaf stage | No tolerance |
| 2015 | WA | Mt Barker | NUSEEDGT50 | Jockey | No tolerance |
| 2015 | WA | Munglinup | THUMPERTT | Jockey, Impact in Furrow, Prosaro @ 375ml/ha @ 4-6 leaf stage | No tolerance |
| 2015 | WA | Munglinup | THUMPERTT | Jockey, Prosaro @ 450ml/ha @ 2-4 leaf stage | Tolerance |
| 2015 | WA | Munglinup | THUMPERTT | Jockey, Prosaro @ 450ml/ha post 4-6 leaf stage | No tolerance |
| 2015 | WA | NE Nyabing | ATRSTINGRAY | Impact in Furrow | No tolerance |
| 2015 | WA | Nyabing | CRUSHERTT | Jockey, Impact in Furrow | Low tolerance |
| 2015 | WA | Ongerup | ATRGEM | Impact in Furrow | No tolerance |
| 2015 | WA | Pingelly | ATRBONITO | Maxim, Impact in Furrow, Prosaro @ 450ml/ha @ 4-6 leaf stage | No tolerance |
| 2015 | WA | Pingelly | CRUSHERTT | Impact in Furrow, Prosaro @ 450ml/ha @ 4-6 leaf stage | No tolerance |
| 2015 | WA | Pingelly | ATRSTINGRAY | Impact in Furrow | No tolerance |
| 2015 | WA | Pingelly | ATRBONITO | Maxim, Impact in Furrow | No tolerance |
| 2015 | WA | Pingelly | CRUSHERTT | Impact in Furrow | No tolerance |
| 2015 | WA | Pingelly | ATRSTINGRAY | Impact in Furrow | No tolerance |
| 2015 | WA | Scaddan | ATRSTINGRAY | None | No tolerance |
| 2015 | WA | South Sterling | ATRGEM | Jockey | Low tolerance |
| 2015 | WA | South Stirling | ATRMARLIN | None | No tolerance |
| 2015 | WA | Williams | CBTELFER | None | No tolerance |
