# Supplementary material for: Identification of isolates of the plant pathogen Leptosphaeria maculans with resistance to the triazole fungicide fluquinconazole using a novel In Planta assay
Source: PLoS One. 2017 Nov 15;12(11):e0188106. doi: 10.1371/journal.pone.0188106 (PMC5687775; doi:10.1371/journal.pone.0188106)
Supplement: S2 Table — (DOCX) [file pone.0188106.s002.docx]

**S2 Table**. **Fungicide sensitive isolates used in this study**

| Isolate name | Synonyms | Year collected | Location | Reference |
| --- | --- | --- | --- | --- |
| D2 | IBCN15 | 1988 | Streatham, Victoria | Marcroft et al 2012 |
| D3 | IBCN16 | 1988 | Mt Barker, Western Australia | Marcroft et al 2012 |
| D4 | IBCN17 | 1988 | Millicent, South Australia | Marcroft et al 2012 |
| D5 | IBCN18 | 1988 | Penshurt, Victoria | Marcroft et al 2012 |
| D6 | IBCN75 | 1987 | Mt Barker, Western Australia | Marcroft et al 2012 |
| D7 | IBCN76 | 1987 | Mt Barker, Western Austrlaia | Marcroft et al 2012 |
| D8 | 05MGPS048 | 2005 | Yeelana, South Australia | Marcroft et al 2012 |
| D9 | 05MGPS049 | 2005 | Yeelana, South Australia | Marcroft et al 2012 |
| D10 | PHW1223 | 1987 | Mt Barker, Western Australia | Marcroft et al 2012 |
| D13 | 09SMW024 | 2009 | Cummins, South Australia | Marcroft et al 2012 |
| D14 | 10SMJ023 | 2010 | Bordertown, Victoria | Marcroft et al 2012 |
| D16 | 13W016 | 2013 | Wangary, South Austrlaia | This study |
| D17 | 13P073 | 2013 | Wangary, South Australia | This study |
| 15FRG066 | N/A | 2015 | Muresk, Western Australia | This study |
